# Effect of straw retention on carbon footprint under different cropping sequences in Northeast China

**DOI:** 10.1007/s11356-021-14316-4

**Published:** 2021-05-20

**Authors:** Qiulai Song, Jie Zhu, Zhenping Gong, Yanjiang Feng, Qi Wang, Yu Sun, Xiannan Zeng, Yongcai Lai

**Affiliations:** 1 Institute of Crop Cultivation and Tillage, Heilongjiang Academy of Agricultural Sciences, Harbin, 150086 Heilongjiang China; 2 Key Laboratory for Combining Farming and Animal Husbandry, Ministry of Agriculture and Rural Affairs, Harbin, 150086 Heilongjiang China; 3 Beijing Chalk Blue Sky Technology Co., Ltd, Beijing, 100102 China; 4 College of Agriculture, Northeast Agricultural University, Harbin, 150030 China

**Keywords:** Straw retention, Continuous corn, Corn-soybean rotation, Carbon footprint, Forming factors, Carbon balance

## Abstract

Inappropriate farm management practices can lead to increased agricultural inputs and changes in atmospheric greenhouse gas (GHG) emissions, impacting climate change. This study was initiated in 2012 to assess the potential for straw retention to mitigate the negative environmental impact of various cropping systems on the Songnen Plain using the life cycle assessment (LCA) method combined with field survey data. Straw retention (STR) and straw removal (STM) treatments were established in continuous corn (CC) and corn-soybean rotation (CS) systems in a split-plot experiment. The effects of straw retention on the carbon footprint (CF) of cropland under different cropping systems were compared. The CF under CC was 2434–2707 kg CO_2_ ha^−1^ year^−1^, 49–57% higher than that under CS. Nitrogen fertilizer produced the most CO_2_, accounting for 66–80% of the CF. The carbon balances of the CC and CS systems with STR were positive, with annual carbon sequestrations of 9633 and 2716 kg CO_2_ ha^−1^ year^−1^, respectively. The carbon balance (CB) of CC-STR was 255% higher than that of CS-STR. This study demonstrates that STR under CC cultivation is an environmentally friendly practice for agricultural production, can help achieve high-yield and low-carbon production in rainfed cropland, and can support the sustainable development of grain production in Northeast China.

## Introduction

Greenhouse gas (GHG) emissions are the most critical factors influencing global climate change, and climate change poses a serious threat to the natural environment and human economic development (IPCC 2013). Agricultural ecosystem is the primary source of GHGs released by human activity (Bennetzen et al. 2016; Linquist et al. 2012). Various cropland management practices affect the mineralization of soil organic matter and alter carbon emissions. Moreover, differences inputs of chemical fertilizers, human activities, and fuels create variation in carbon emissions from agricultural inputs under different management practices, indirectly influencing the energy consumption and carbon cycling of systems (Li et al. 2002; Lal 2004; Larsen and Hertwich 2011; Wang et al. 2015a; Zhang et al. 2015; Meier et al. 2020). The carbon footprint (CF), the impact of carbon emissions on the global environment, is an assessment of direct or indirect CO_2_ emissions caused by particular activities or estimated cumulatively during the life cycles of particular products (Peters 2010; Duan et al. 2011; Adewale et al. 2019). The factors influencing CFs include the CO_2_ emissions from farmland soil and crops and indirect CO_2_ emissions from the production, storage, and transportation of agricultural production materials (Liu et al. 2016; Lal et al. 2019).

CFs are affected by many factors, such as regional conditions, agricultural production systems, and crop types (Günther et al. 2017; Houshyar and Grundmann 2017; Yadav et al. 2017; Liu et al. 2018; Xue et al. 2018). To quantify the CFs of different agricultural production systems around the world, many studies of regional agricultural CFs, crop CFs, and food CFs have been conducted (Hillier et al. 2009a; Nelson et al. 2009; Wang et al. 2015b). Previous studies have quantified the CFs of different crops and patterns of variation in different regions (Hillier et al. 2009b; Röös et al. 2010; Clay et al. 2012; Gan et al. 2014; Wang et al. 2015a, 2016; Günther et al. 2017; Houshyar and Grundmann 2017; Pishgar-Komleh et al. 2017; Yadav et al. 2017; Heusala et al. 2020), providing a basis for reducing carbon emissions in agricultural production processes. The CF of crop production can be reduced by changing management methods and implementing low-carbon technologies, such as conservation tillage, optimized irrigation, and fertilizer application (Zhang et al. 2016; Yadav et al. 2018). Wang et al. (2020) assessed the CFs of four different cropping systems, and the results indicated that cotton monoculture was the best (i.e., had the lowest CF) of these cropping systems in low-fertility plots and that winter wheat intercropped with cotton was best (i.e., had the lowest CF) in high-fertility plots.

Straw retention (STR) also has an important influence on the CF. Lal et al. (2019) demonstrated that STR increased CFs by approximately 10%. Li et al. (2020) further pointed out that the CF is strongly affected by the amount of straw used, and when compared with no STR treatment, the CF did not increase until field application of one-third of the STR and then increased as straw application was further increased. Bai et al. (2021), under the same natural conditions in semiarid areas of Northwest China, showed that STR increased GHG emissions, but the CF decreased by 45–55% due to the strong acceleration of soil organic carbon (SOC) accumulation. Therefore, the effects of STR on the CF observed by different researchers in different regions are inconsistent. These studies have systematically elucidated the impacts of crop rotation on CFs as well as the responses of soil carbon emissions and CF to farming practices, including STR. However, little has been reported on how the combined effects of crop rotation and STR affect the CF.

The Songnen Plain is a major grain-producing area in Northeast China. This plain region is located in Heilongjiang and Jilin Provinces. Rainfed cropland in this region is mainly planted with corn and soybean. The cropping system involves one harvest per year, and the major cropping patterns are continuous corn (CC) and corn-soybean rotation (CS). In recent years, the Chinese government has completely prohibited burning crop straw in the field and has vigorously promoted straw return technology. The area of crop straw return has increased year-by-year in the Songnen Plain. However, there has been no systematic study of the effects of STR on the CF under these two cropping patterns (CC and CS) on the Songnen Plain. We hypothesized that CFs are jointly influenced by differences in cropping pattern (CC or CS) and straw-use pattern, e.g., STR or straw removal (STM). Our objective was to use life cycle assessment (LCA) to evaluate the impact of STR on CFs under two cropping patterns (CC and CS) on the Songnen Plain through direct measurement of soil carbon emissions and indirect emission inventories.

## Materials and methods

### Experimental site

The field experiment was conducted at the Xiangfang Experimental Practice Base of Northeast Agricultural University. During the experimental period, the total annual rainfall was 485 mm (2013) and 454 mm (2014). This study began in 2012, and data were collected from 2013 to 2014. The cropping patterns at the experimental site were mainly CC and CS. The cropping sequence of CC was corn in both 2013 and 2014, and the cropping sequence of CS was corn in 2013 followed by soybean in 2014. The basic soil physicochemical properties (0–20 cm depth) are listed in Table 1.

**Table 1:** Principal chemical properties of the experimental soil

Soil depth (cm)	Organic matter (g kg^−1^)	Total nitrogen (g kg^−1^)	Total phosphorus (g kg^−1^)	Total potassium (g kg^−1^)	NO_3_^−^-N (mg kg^−1^)	NH_4_^+^-N (mg kg^−1^)	Available phosphorus (mg kg^−1^)	Available potassium (mg kg^−1^)
0–20	30.71	1.48	0.40	16.28	78.79	26.04	23.63	187.00

### Experimental design

A two-factor split-plot design was used in this study. The main plot factor was cropping pattern (CC vs. CS), and the subplot factor was straw management (STR vs. STM). There were four treatments: continuous corn cropping with straw retention (CC-STR), continuous corn cropping with straw removal (CC-STM), corn-soybean rotation with straw retention (CS-STR), and corn-soybean rotation with straw removal (CS-STM). Each treatment had three replicates for a total of 12 plots, with 780 m^2^ per plot.

In the STR treatment, the corn straw was cut into pieces (≤10 cm) after harvest in autumn and returned to the field. A ridge subsoiling stubble machine was used to deep-loosen the soil to 25 cm and form a seeding strip of 32 cm. In the STM treatment, the straw was removed from the field after harvest, the stubble and soil were plowed to a depth of 25 cm, and a rotary cultivation machine was used to crush the soil and ridge at the same time.

In all four treatments, the ridge spacing was 70 cm. During the crop seedling stage, the soil was cultivated with medium tillage.

During the 2-year experimental period, the same crop cultivar, fertilization, and weeding schemes were used, and the corn and soybean were sown and harvested at the same time. The Dongnong 253 corn (*Zea mays* L.) cultivar was sown mechanically on May 2 and harvested on October 6, with a mean density of 65,000 plants ha^−1^. The specific rates of fertilizer application for corn were as follows: urea (46% N), 300 kg ha^−1^ (75 kg ha^−1^ sowing and 225 kg ha^−1^ topdressing); diammonium phosphate (18% N and 46% P_2_O_5_), 150 kg ha^−1^; and potassium sulfate (30% K_2_O), 75 kg ha^−1^. The Kenfeng 16 soybean (*Glycine max*) cultivar was mechanically sown on May 2 and harvested on September 28 with a seeding rate of 43.66 kg ha^−1^ and a mean density of 269,500 plants ha^−1^. The rates of fertilizer application for soybean were as follows: diammonium phosphate (18% N and 46% P_2_O_5_), 150 kg ha^−1^, and potassium sulfate (30% K_2_O), 75 kg ha^−1^. For chemical weeding, 96% emulsifiable concentrate of Dual Gold mixed with 72% emulsifiable concentrate of 2,4-D butyl ester was applied for closed weed control 1 week after sowing of corn and soybean, with dosages of 975 ml ha^−1^ and 1125 ml ha^−1^, respectively. In addition, 55% Gengjie was sprayed at the four-to-five leaf stage of corn at a dosage of 1575 ml ha^−1^, and 36% fomesafen-quizalofop-*p*-ethyl-clomazone was sprayed on soybean plants after the development of one to three compound leaves at a dosage of 1650 ml ha^−1^.

### Calculation of cropland CF

**Fig. 1:**
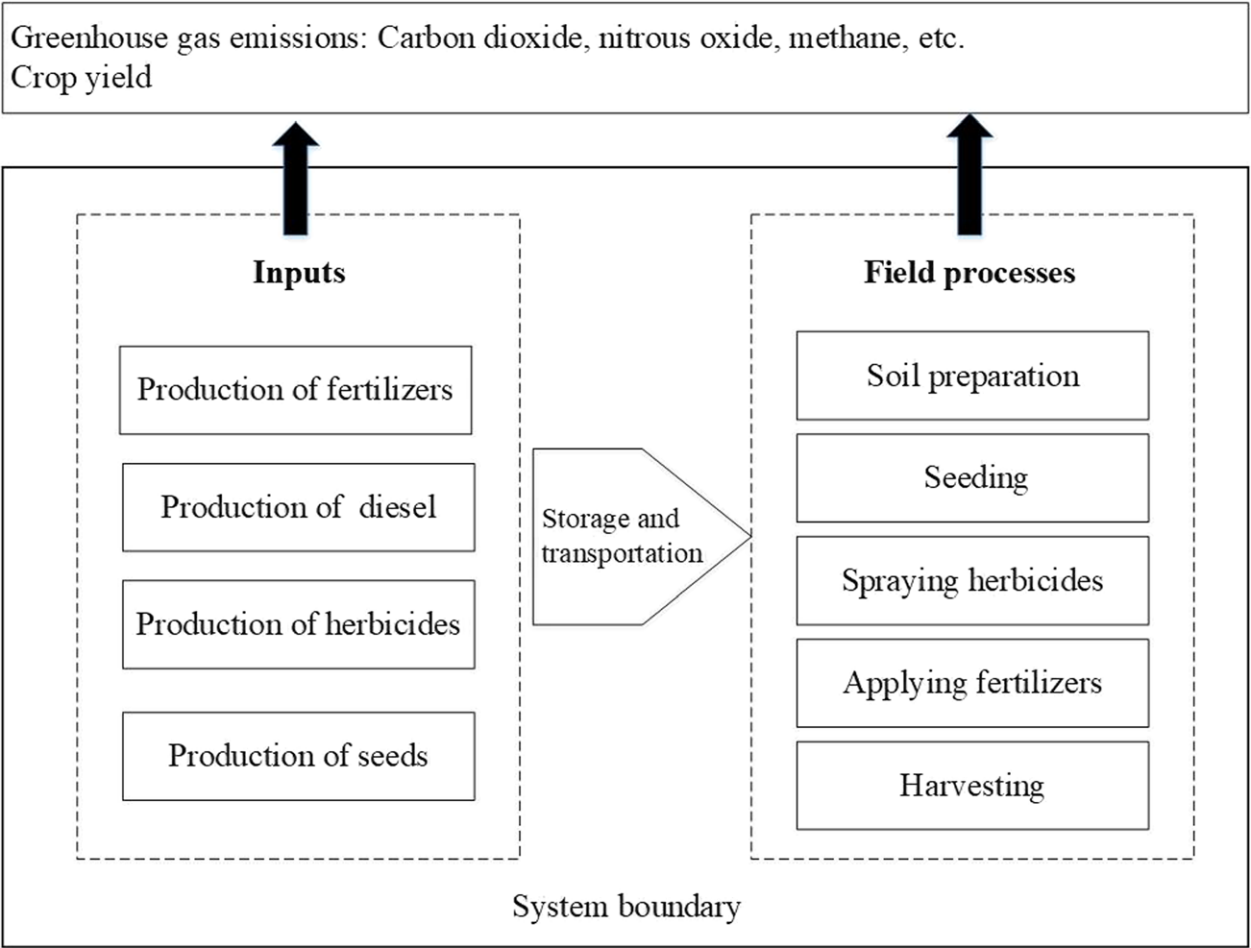
System boundary for calculating GHG emissions in continuous corn cropping and corn-soybean rotation cropping systems

The system boundary of cropland CF was determined following the principles of LCA (Mohammadi et al. 2013), as shown schematically in Fig. 1. The carbon flux changes of the elements in the carbon cycle of the system were determined and calculated according to the CF equation developed by Liu et al. (2013); She et al. (2017); and Feng et al. (2020). The CF was calculated as follows:

1 $$ CF={GWP}_{N_2O}+{GWP}_{input} $$

where CF is the total carbon emissions of crop production, ${\mathrm{GWP}}_{{\mathrm{N}}_2\mathrm{O}}$ is the total emissions produced by synthetic nitrogen fertilizer and crop residual nitrogen (kg CO_2_ ha^−1^ year^−1^), and GWP_input_ is the indirect GHG emissions from the production, storage, transportation, and use of agricultural inputs.

${\mathrm{GWP}}_{{\mathrm{N}}_2\mathrm{O}}$ emissions were estimated based on the levels of synthetic nitrogen fertilizer and crop residual nitrogen by the method determined by the IPCC (2019). ${\mathrm{GWP}}_{{\mathrm{N}}_2\mathrm{O}}$ emissions were calculated as follows:

2 $$ {\mathrm{GWP}}_{{\mathrm{N}}_2\mathrm{O}}={GWP}_{N_2{O}_{SNF}}+{GWP}_{N_2{O}_{CRN}} $$

3 $$ {\mathrm{GWP}}_{N_2{O}_{SNF}}={Q}_{SNF}\times \left[ EF+\left({F}_{\mathrm{volatilization}}\times {\mathrm{E}}_{\mathrm{volatilization}}\right)+\left({F}_{\mathrm{leach}}\times {\mathrm{E}}_{\mathrm{leach}}\right)\right]\times 44/28\times 298 $$

4 $$ {\mathrm{GWP}}_{{\mathrm{N}}_2\mathrm{O}}={Q}_{CRN}\times \left[ EF+\left({F}_{\mathrm{leach}}\times {\mathrm{E}}_{\mathrm{leach}}\right)\right]\times 44/28\times 298 $$

where ${\mathrm{GWP}}_{N_2{O}_{SNF}}$ represents N_2_O emissions from farmland resulting from synthetic nitrogen fertilizer application (kg CO_2_ ha^−1^ year^−1^), ${\mathrm{GWP}}_{N_2{O}_{CRN}}$ represents N_2_O emissions from crop residual nitrogen (kg CO_2_ ha^−1^ year^−1^), *Q*_*SNF*_ represents the amount of synthetic nitrogen fertilizer (kg N ha^−1^ year^−1^), *Q*_*CRN*_ represents the crop residue nitrogen (kg N ha^−1^ year^−1^), EF is the direct emission factor (kg N_2_O-N/kg N, 0.01), F_volatilization_ is the rate of volatilization of synthetic nitrogen fertilizer as NH_3_-N and NO_x_-N (15%), E_volatilization_ is the emission factor for N_2_O volatilized as NH_3_-N and NO_x_-N (0.014), F_leach_ is the percent nitrogen loss via nitrate leaching and runoff in the total nitrogen input (24%), E_leach_ is the emission factor for N_2_O from nitrate leaching (0.011), 44/28 is the conversion factor for N_2_O-N to N_2_O, and 298 is the global warming potential of N_2_O over a 100-year period (Yang et al. 2014; IPCC 2019; Wang et al. 2020).

GWP_input_ is the CO_2_ emissions from agricultural inputs during agricultural production, calculated as follows:

5 $$ {\mathrm{GWP}}_{\mathrm{i}\mathrm{nput}}={\sum}_{\mathrm{i}=1}^{\mathrm{n}}{\mathrm{AL}}_{\mathrm{i}}\times {\mathrm{EF}}_{\mathrm{i}} $$

where AL_i_ is the ith input variable and EF_i_ is the emission factor for the ith input variable. The emission factors were mainly derived from Liu et al. (2013) and Yang et al. (2014) (Table 2). Specifically, diesel input was determined by measuring diesel fuel consumption during soil preparation, seeding, intertillage, and harvesting using a multifunction fuel consumption meter (Shuangshuo Electronics Co., Ltd., Zibo, Shandong Province, China). The measurement was performed on a row length of 100 m and repeated three times. Agricultural chemical inputs were calculated as the amounts of chemical elements according to the inputs reported in the “Experimental design” subsection, and the agricultural inputs are listed in Table 3.

**Table 2:** Emission factors for agriculture inputs used in the estimation

	(kg CO_2_ ha^−1^ year^−1^)	Reference
N	4.96	(Liu et al. 2013)
P	1.14	(Liu et al. 2013)
K	0.66	(Liu et al. 2013)
Herbicide	6.58	(Liu et al. 2013)
Corn seeds	1.22	(Liu et al. 2013)
Soybean seeds	0.92	(West and Marland 2002)
Diesel	3.32	(Liu et al. 2013)

**Table 3:** Average agricultural inputs for crops (kg ha^−1^ year^−1^)

		Corn		Soybean	
		Straw retention	Straw removal	Straw retention	Straw removal
Diesel	Soil preparation	8.57	25.35	8.57	25.35
	Seeding	5.09	6.26	5.47	5.49
	Spraying herbicides	1.70	2.09	1.82	1.83
	Intertillage	8.88	9.23	9.16	9.52
	Topdressing	5.09	6.26		
	Harvest	25.00	25.00	20.00	20.00
Agricultural inputs	N	165.00	165.00	27.00	27.00
	P	69.00	69.00	69.00	69.00
	K	22.50	22.50	22.50	22.50
	Herbicide	2.61	2.61	2.30	2.30
	Seeds	18.75	18.75	43.66	43.66

### Calculation of cropland carbon balance

Net biome productivity (NBP) is the change in net carbon storage of the cropland ecosystem, calculated as follows (Huang et al. 2013; She et al. 2017):

6 $$ NBP= NPP- CR-{R}_s $$

where NPP is net primary productivity, CR is the grain and straw removed with crop harvest, and R_s_ is the heterotrophic soil microbial respiration. NPP includes carbon sequestered by crop grains, straw, and roots. NPP was calculated from measurements of the grain yield at harvest, the dry weight percentages of plant parts, and the carbon content measured in various parts of the plants. CR includes crop grains, stalks, and cobs removed from the field after harvest. Under the STR treatment, only the corn and soybean grains were harvested from the field, while under STM, corn grains, cobs, and stalks and soybean grains, pods, and stalks were all harvested from the field. R_s_ was estimated from the actual field measurement of total soil in situ respiration according to the ratio of heterotrophic respiration to total in situ respiration for the same area as reported by Zhu (2015) (65% for corn and 76% for soybean) (Table 4).

**Table 4:** Yield, biomass, and NPP under different modes

Agricultural system		Yield (kg ha^−1^)^a^		Biomass (kg ha^−1^)^b^		NPP (kg C ha^−1^)^c^	
Year	Crop	Straw retention	Straw removal	Straw retention	Straw removal	Straw retention	Straw removal
2013	Corn	9185b	10,624a	20,231	23,402	9534	11,028
2014	Corn	13,426a	11,948a	29,572	26,316	13,935	12,401
2014	Soybean	2275b	2938a	6438	8314	2760	3563
Continuous corn croppingd		11,305	11,286	24,901	24,859	11,734	11,714
Corn-soybean rotationd		5730	6781	13,335	15,858	6147	7295

^a^Differing lowercase letters for horizontal comparisons indicate significant differences between the treatments for the same crop (*p* < 0.05)

^b^Biomass of corn and soybean converted from grain yield. The dry-weight percentages of various parts of the corn plants were as follows: grains, 45.4%; roots, 9.4%; stalks, 38.3%; and cobs, 6.9%. The dry-weight percentages of various parts of the soybean plants were as follows: grains, 35.3%; roots, 5.2%; stalks, 18.6%; petioles, 25.0%; and pod walls, 15.8%

^c^NPP was calculated from the grain yield and the dry weight percentages and carbon contents of various parts of corn and soybean plants. The carbon contents of various parts of corn plants were as follows: grains, 48.9%; roots, 46.5%; stalks, 45.3%; and cobs, 46.4%. The carbon contents of various parts of soybean plants were as follows: grains, 42.6%; roots, 46.8%; stalks, 47.9%; petioles, 38.0%; and pod walls, 43.9%

^d^The grain yield, biomass, and NPP for continuous corn cropping are mean values for corn in 2013 and 2014. The grain yield, biomass, and NPP for the corn-soybean rotation are the mean values for corn in 2013 and for soybeans in 2014

The total soil in situ respiration was measured using the static box-infrared gas analyzer method. Gas samples were collected every 7 to 10 days from April 5 to November 8. Sampling boxes were made of stainless steel, 50 cm long, 25 cm wide, and 50 cm high. Gas samples were collected between 8:30 and 10:30 am on sunny days. Five sampling sites were randomly selected in the treatment plots. Sampling boxes were inserted between two ridges and sealed with approximately 5 cm of soil, and gas was then transferred into 500-ml aluminum foil bags using a 100-ml glass syringe. The CO_2_ concentration was determined using a GXH-3010E1 infrared analyzer (Institute of Beijing HUAYUN Analytical Instrument Co., Ltd.).

The CB of cropland was used to indicate the difference between the CF and NBP as follows:

7 $$ CB= NBP- CF $$

### Statistical analysis

The data were analyzed using descriptive statistics in Microsoft Excel 2016 (Microsoft Corp., Redmond, WA, USA) and IBM SPSS 19.0 (SPSS Inc., Chicago, IL, USA). The results included the means and standard deviations (SD) of three replicates, and Duncan’s multiple range test was used at a significance level of *P*<0.05.

## Results and analysis

### CF of cropland under different cropping patterns

**Fig. 2:**
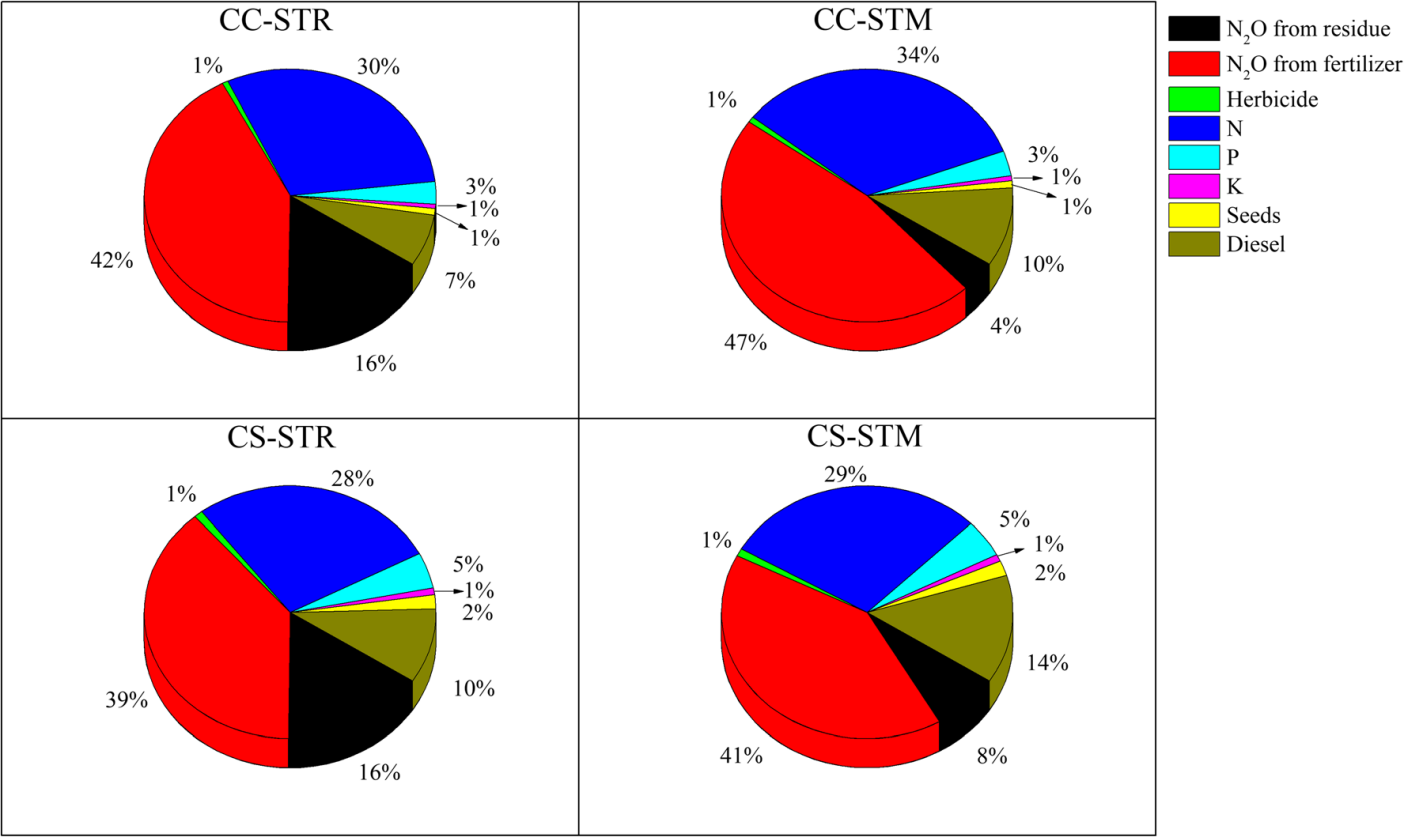
Shares of different inputs in the carbon footprints of continuous corn cropping and corn-soybean rotation cropping systems (two-season averages)

The CO_2_-equivalent emissions estimated based on N_2_O produced by nitrogen fertilizer and straw application were the greatest contributors to the CF (Fig. 2). The percentage of direct N_2_O emissions to total emissions was as follows: 58% for CC-STR, 51% for CC-STM, 55% for CS-STR, and 48% for CS-STM. STR resulted in higher N_2_O emissions from both the CC and CS systems. The second greatest contributor was indirect CO_2_ emissions from the production, storage, and transportation of nitrogen fertilizer, accounting for 30% and 34% of total emissions from CC and 28% and 29% of total emissions from CS. In addition, diesel consumption by agricultural machinery operations from sowing to harvesting produced considerable carbon emissions. In both CC and CS, carbon emissions from diesel consumption were higher under STM (226–246 kg CO_2_ ha^−1^ year^−1^) than under STR (165–180 kg CO_2_ ha^−1^ year^−1^).

The CF of CC was higher than that of CS (Table 5). Due to the large amount of nitrogen in crop straw, the CF of CC with STR (2707 kg CO_2_ ha^−1^ year^−1^) was 11% higher than that of CC with STM (2434 kg CO_2_ ha^−1^ year^−1^) and 6% higher under CS.

**Table 5:** CF, NBP, and CB of cropland under different cropping patterns

Agricultural system		CF (kg CO_2_ ha^−1^ year^−1^)		NBP (kg CO_2_ ha^−1^ year^−1^)		CB^a^ (kg CO_2_ ha^−1^ year^−1^)	
Year	Crop	Straw retention	Straw removal	Straw retention	Straw removal	Straw retention	Straw removal
2013	Corn	2625	2428	8697	−2351	6071	−4780
2014	Corn	2789	2440	15,982	41	13,193	−2399
2014	Soybean	816	832	176	−401	−639	−1233
Continuous corn cropping^b^		2707	2434	12,339	−1155	9633	−3589
Corn-soybean rotationb		1721	1630	4436	−1376	2716	−3006

^a^A positive value for CB indicates that the system is a sink for atmospheric CO_2_, while a negative value for CB indicates that the system is a source for atmospheric CO_2_

^b^CF, NBP, and CB for continuous corn cropping are the mean values for corn in 2013 and 2014. The CF, NBP, and CB for corn-soybean rotation are the mean values for corn in 2013 and for soybeans in 2014

### Soil heterotrophic respiration under different cropping patterns

**Fig. 3:**
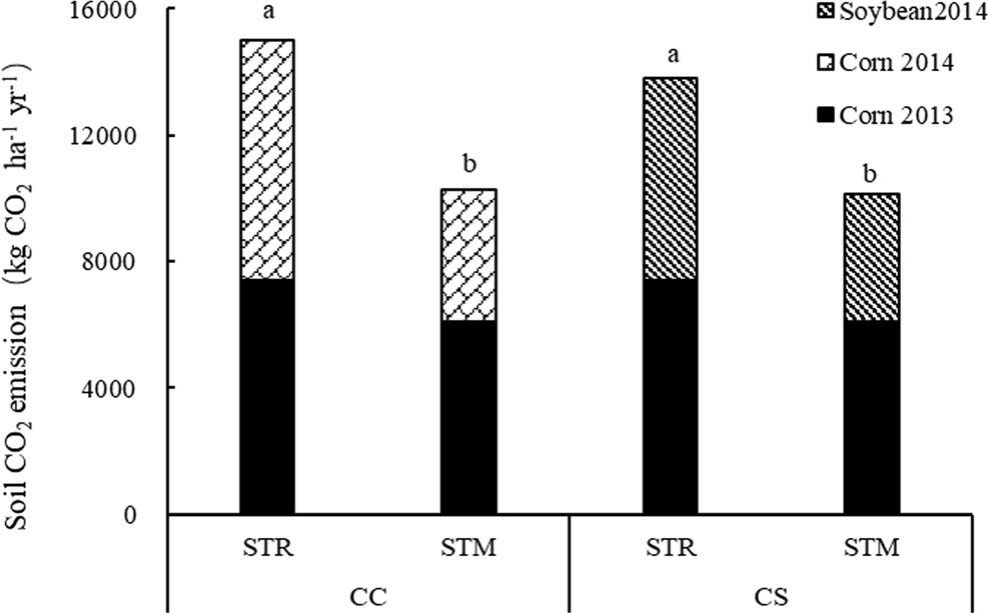
Soil heterotrophic respiration under different cropping patterns

The total soil heterotrophic respiration of CC was similar to that of CS (Fig. 3). Total emissions ranged from 5139 to 7493 kg CO_2_ eq ha^−1^ year^−1^ under CC and from 5072 to 6902 kg CO_2_ eq ha^−1^ year^−1^ under CS. STR significantly increased total heterotrophic respiration by 46% under CC and 36% under CS compared with STM (*p*<0.05).

### NPP under different treatments

Corn with higher grain yield produced more biomass and NPP than did soybean, leading to differences in yield, biomass, and NPP under different cropping patterns. CC produced significantly higher crop yields than CS. STR significantly reduced soybean yield, while its effect on corn yield was not significant compared with that of STM. Overall, STR resulted in lower values for yield, biomass, and NPP in the CS system (Table 4).

### CB of cropland under different cropping patterns

The NBP of the CC and CS systems with STR were 12,339 and 4436 kg CO_2_ ha^−1^ year^−1^, respectively, and the NBP of CC-STR was 178% higher than that of CS-STR. The CB of cropland was also positive, with annual carbon sequestrations of 9633 and 2716 kg CO_2_ ha^−1^ year^−1^, respectively. The CB of CC-STR was 225% higher than that of CS-STR. In contrast, NBP was negative for the CC and CS systems with STM, with values of −1155 and −1376 kg CO_2_ ha^−1^ year^−1^, respectively. For CO_2_-equivalents from soil N_2_O and agricultural inputs, there were strong GHG emissions effects, with annual releases of −3589 and −3006 kg CO_2_-equivalents ha^−1^ year^−1^, respectively. These results indicate that straw retention plays a significant role in carbon sequestration under both the CC and CS systems.

## Discussion

### Variations in CF under different cropping patterns

Inputs and outputs of agricultural ecosystems vary with cropping pattern, leading to differences in CF (Gan et al. 2012; Yang et al. 2014; Wang et al. 2020). Our study also obtained similar results; the inputs of nitrogen fertilizer, diesel fuel, and straw were higher for CC than for CS, resulting in a higher CF with CC than with CS. Similar results were reported by Yadav et al. (2018) and Lal et al. (2019).

N_2_O emissions were the greatest contributor to the total CF, followed by indirect N_2_O emissions from nitrogen fertilizer production, storage, and transportation. This result agrees with the findings of Yadav et al. (2018). However, Jat et al. (2019) and Lal et al. (2019) reported that fertilizer application makes the greatest contribution, followed by N_2_O emissions and diesel emissions. Our findings were not entirely consistent with these results. These conflicting results may be explained by noting that Jat et al. (2019) and Lal et al. (2019) did not consider N_2_O volatilization and leaching.

Despite differences in these studies, they all demonstrate that indirect N_2_O emissions from the production, storage, and transportation of nitrogen fertilizer as well as direct N_2_O emissions from the application of nitrogen fertilizer are the most important components of total GHG emissions from crop production (Hillier et al. 2009a; Cheng et al. 2011; West et al. 2014; Wang et al. 2020). Therefore, reducing nitrogen fertilizer input and adopting a sustainable application method are crucial practices to mitigate agricultural GHG emissions from fertilizer application (Bacenetti et al. 2016; Feng et al. 2020). It should be noted that reducing nitrogen fertilizer may affect yield and that the amount of nitrogen fertilizer should be adjusted by comprehensively considering CF changes per unit of yield. In this study, diesel input was the third highest contributor to the CF (7–14%). During soil preparation, minimal tillage and no-tillage with reduced agricultural machinery operation can reduce GHG emissions (Yadav et al. 2018).

### Carbon balance of cropland under different cropping patterns

Carbon sequestration and carbon emissions are two processes that coexist in agricultural production. GHGs such as CO_2_ and N_2_O are directly or indirectly emitted into the atmosphere, while plants absorb atmospheric CO_2_ through photosynthesis (Soussana et al. 2007; Smith et al. 2010; Liu et al. 2018; Feng et al. 2020). The CB of cropland can directly characterize changes in net carbon flow in cropland systems (Feng et al. 2020). Generally, if all crop straw is returned to the farmland, then it is equivalent to the amount of GHG released after the straw is decomposed. Therefore, neither straw carbon sequestration nor straw carbon emissions are considered in general (Feng et al. 2020). However, our study aimed to assess the effects of STR and STM on the CB of cropland under two different cropping patterns; thus, crop straw inputs were considered. Although this approach may exaggerate the carbon sequestration effect of STR, the carbon sequestration trend was clear. Huang et al. (2019) obtained CFs based on changes in soil organic carbon storage in Jilin Province, showing that net carbon sequestration was 745 kg CO_2_ ha^−1^ year^−1^ under CC with minimal tillage and STR. In our study, following straw retention, the carbon sequestered by CC was 9633 kg CO_2_ ha^−1^ year^−1^, and the carbon sequestered by CS was 2716 kg CO_2_ ha^−1^ year^−1^. The carbon sequestration of CC reported here is higher than that reported by Huang et al. (2019), but this result may reflect the carbon sequestration effect of straw return. Due to differences in study methods and boundaries, discrepancies exist in results obtained from the same region by different researchers, but the data all reflect the advantage of straw retention for carbon sequestration. Lemke et al. (2010) and Huang et al. (2019) reported that if there is not enough crop straw to return, cropland soil will become a CO_2_ source. Our study reaches a similar conclusion. Both cropping patterns were a source of atmospheric CO_2_ under STM.

### Limitations and implications of this study

This study ignores GHG emissions from agricultural labor and agricultural machinery manufacturing, transportation, maintenance, and management. From the life cycle perspective, these GHG emissions are not negligible (Liu et al. 2013). If these factors are considered in CF calculations, the absolute value of the CF may change. This study compared the effects of differences in planting pattern and straw utilization on the CF to determine the most favorable planting pattern rather than obtaining absolute values for the CF of planting patterns. Although the calculation method employed in this paper requires improvement, it can provide a basis for further research and guide low-carbon agricultural production and is relevant to national carbon emission and environmental impact assessments.

## Conclusions

STR greatly impacted GHG emissions, CF, and CB. The CF was higher for CC than for CS, and nitrogen fertilizer was the most important factor affecting the CF. When considering the carbon fixed by crops, the CB of the STR treatment was positive for CC and CS. Crop yield and CB were higher in CC-STR than in CS-STR. Straw return in CC can promote high yield and low carbon emissions, provide improved ecological benefits, and accelerate clean and sustainable production in the Songnen Plain of Northeast China.

## Data Availability

The datasets used and/or analyzed during the current study are available from the corresponding author on reasonable request. All data generated or analyzed during this study are included in this published article and its supplementary information files.
